# Preductal Segmental Tubular Aortic Hypoplasia in Perinatally Died Stabyhoun Puppies

**DOI:** 10.3390/ani13152423

**Published:** 2023-07-27

**Authors:** Marie D. B. van Staveren, Guy C. M. Grinwis, Marjolein L. den Toom, Viktor Szatmári

**Affiliations:** 1 Department Clinical Sciences, Faculty of Veterinary Medicine, Utrecht University, 3584 CM Utrecht, The Netherlands; 2 Department Biomolecular Health Sciences, Faculty of Veterinary Medicine, Utrecht University, 3584 CL Utrecht, The Netherlands

**Keywords:** coarctation, congenital, dogs, ductus arteriosus, hereditary, necropsy, neonatal, PDA, perinatal, stabij

## Abstract

**Simple Summary:**

Because of an unexplained rise in the death rate of newborn puppies of the Stabyhoun breed, one of the Dutch breeders’ organizations contacted the reporting institution for help. Consequently, eight Stabyhoun puppies that were stillborn, died or were euthanized within weeks after birth were submitted to post-mortem examination at the reporting institution over a period of 18 months. In five of the eight Stabyhoun puppies, the aorta, which is the largest artery of the body, showed a severe local narrowing in the chest. Narrowing of the aorta in dogs has extremely rarely been documented. Therefore, the relatively high rate of occurrence in these eight examined Stabyhoun puppies is an unexpected finding, which might have been caused by a genetic predisposition. In children, narrowing of the aorta is believed to develop as a result of constriction of an abnormally located muscle in the vascular wall at birth. If this constriction cannot happen before birth, this vascular anomaly cannot explain the death of the stillborn puppies. However, the condition might lead to heart failure and other clinical signs later in life. Because the aorta is not routinely examined at post-mortem examinations, the frequency of this anomaly in puppies is unknown.

**Abstract:**

Background: A high perinatal mortality rate in the Stabyhoun breed prompted one of the Dutch breeding organizations to start an investigation. Preductal segmental tubular aortic hypoplasia is an extremely rarely documented congenital vascular anomaly in dogs, and it is suspected to be the result of constriction of ectopic ductal tissue in the aortic wall at birth. Methods: Over a period of 18 months, Stabyhoun puppies that were stillborn, died or were euthanized before 3 weeks of age were submitted to post-mortem examination at the reporting institution. Pathologic findings were documented. Results: Eight Stabyhoun puppies were submitted during the study period. In five of them, a severe preductal segmental tubular aortic hypoplasia was found. Two of the five puppies were stillborn, and three died spontaneously or were euthanized. Conclusions: Preductal tubular aortic hypoplasia was found in an unusually high frequency in the examined Stabyhoun puppies. Because the condition is believed to cause clinical signs only after birth, this anomaly cannot explain the death of the stillborn puppies. However, it might be responsible for cardiogenic pulmonary edema in the postnatal period. Routine dissection of the great vessels in perinatally deceased puppies would help to establish the prevalence of congenital anomalies of the aorta.

## 1. Introduction

Patent ductus arteriosus (PDA) is one of the most common congenital cardiovascular diseases in dogs [1]. Failure of postnatal closure of the ductus arteriosus typically leads to left-to-right shunting via the PDA, characterized by varying amounts of blood shunting from the descending aorta to the pulmonary artery [1,2]. If the shunting is hemodynamically relevant, congestive left-sided heart failure and/or pulmonary hypertension develops with high morbidity and mortality rates [2]. The Dutch Stabyhoun breed was reported to have a high prevalence and a genetic predisposition to PDA in a study performed in the Netherlands [3].

Aortic segmental tubular hypoplasia is characterized by a narrowing of one or more anatomic segments of the aorta [4,5,6,7,8,9,10]. Aortic segmental tubular hypoplasia and aortic coarctation have both been documented in animals; however, they are extremely rare, particularly in dogs [11,12,13,14]. Contraction of ectopic ductal tissue in the aortic wall simultaneous with the closure of the ductus arteriosus at birth is believed to be the underlying pathogenesis of aortic coarctation in humans [4,5,6,7,8,9,10,11,12,13,14].

Perinatal mortality is defined as the sum of stillborn puppies and puppies that die in the first week of life [15]. Perinatal mortality has been reported to be present in 25% of litters and affects 5–8% of puppies [15,16]. Congenital malformations are among the possible causes of perinatal mortality, with a reported prevalence of 7% [16]. The most commonly reported congenital malformations are cleft palate and hydrocephalus [15]. However, many congenital anomalies, such as those of the cardiovascular system, may not cause any clinical signs in the first weeks of life, and they cannot be appreciated by inspection alone and not even at routine necropsy [1,2,15,16,17].

The present observational study describes preductal segmental tubular aortic hypoplasia in five Stabyhoun puppies, which were stillborn, died spontaneously or were euthanized before they reached 3 weeks of age.

## 2. Materials and Methods

Because one of the Dutch Stabyhoun breeders’ organizations in the Netherlands had the impression that the perinatal mortality rate increased among the Stabyhoun breed, they reached out to the reporting institution to try to identify the underlying cause. The pathology service of the reporting institution offered to perform post-mortem examinations of stillborn puppies and puppies that died or were euthanized in the perinatal period.

All puppies that were brought to the pathology service were included in the study; no puppies were excluded. The breeders were responsible for bringing the cadavers to the reporting institution within 2 days after death. If transport was not feasible on the day of death, the cadavers were refrigerated at 4 degrees Celsius until transport could be realized. No selection took place regarding which puppies should be brought to pathology. However, the participation of the breeders was voluntary. These factors might have led to some selection bias, as certain breeders did not wish to participate or simply did not have the time or the opportunity to arrange transport of the cadavers to the reporting institution in a short time frame. Post-mortem examinations consisted of gross and focused histopathologic examinations performed at a single institution by the attending pathologist. Comprehensive autopsies were performed by board-certified veterinary pathologists according to standard pathology protocols, in which organs and tissues were analyzed grossly for the absence or presence of both congenital and acquired lesions. For gross evaluation, the heart and large vessels were left attached to the lungs. The aorta was dissected at the diaphragm and opened at the dorsal side from caudal to cranial direction using small scissors to allow for visualization of the ductus arteriosus and assessment of the diameter of the proximal aorta at the site of the ductus. Subsequently, the apex of the heart was removed, the free walls of the left and right ventricles were cut open, and the associated atria were opened using small scissors. After opening of the heart, the ventricular and atrial septa as well as the left and right atrioventricular valves were evaluated. Then, the pulmonary artery was opened, and the vessels and valves were assessed. Finally, the leaflet of the left atrioventricular valve spanning the aortic orifice was cut to allow for evaluation of the aortic valve leaflets and the aortic arch. Since the cardiovascular structures were small, in most cases, surgical loupe spectacles were used to facilitate the gross evaluation of the heart and large vessels. In each puppy, special attention was paid to the patency of the ductus arteriosus because of the high prevalence and the known genetic predisposition for PDA in this breed in the same population [3].

## 3. Results

Between 2016 and 2018, over a period of 18 months, eight Stabyhoun puppies younger than 3 weeks of age were submitted for post-mortem examination to the pathology service of the reporting institution by members of the above-mentioned Dutch Stabyhoun breeders’ organization. In five of these eight puppies, segmental aortic hypoplasia was found (Table 1). The remaining three puppies were stillborn, of which two originated from the same litter. The cause of death of these three female stillborn puppies was thought to be intrauterine asphyxia.

**Figure 1:**
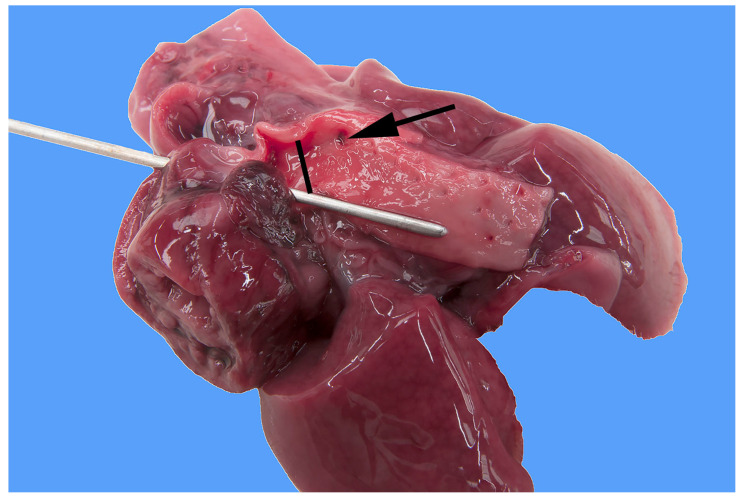
Heart and lung of a stillborn female Stabyhoun puppy (puppy #2) viewed from the left side, where the left of the image is cranial, and the right of the image is caudal. The ventral half of the heart was cut off. The ductus arteriosus, which is patent (indicated by the black bar), and the descending aorta caudal to it are opened, and their lumens are exposed. A metal probe is inserted from the pulmonary artery through the patent ductus arteriosus to the lumen of the descending aorta. The arrow indicates the markedly narrowed lumen of the preductal aortic segment.

**Figure 2:**
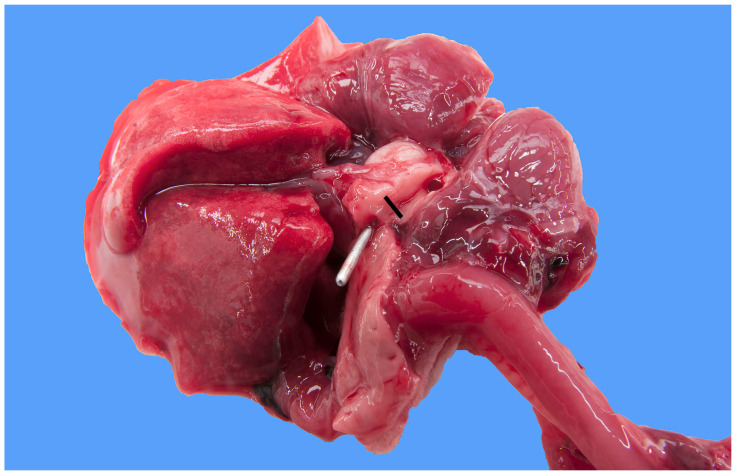
Heart and lung of a 1-day-old male Stabyhoun puppy (puppy #3) viewed from a caudo-dorsal aspect, where the upper right corner of the image is cranially oriented. The ventral half of the heart was cut off. A metal probe is inserted from the pulmonary artery (not visible) through the patent ductus arteriosus to the descending aorta. The lumens of both the pre- and post-ductal segments of the aorta were cut open and are exposed. The diameter of the preductal aorta (indicated by the bar) measured about 3 mm and was much narrower than that of the post-ductal aorta (where the metal probe is located).

**Figure 3:**
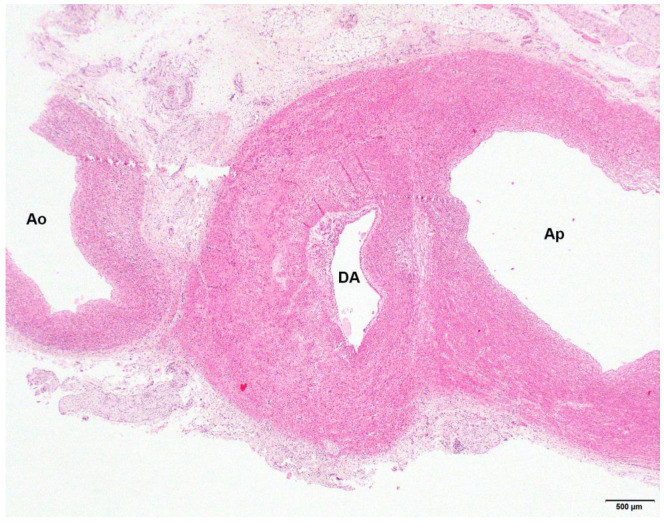
Photomicrograph of the hypoplastic aorta (Ao), the patent ductus arteriosus (DA) and the pulmonary artery (Ap) of a 16-day-old Stabyhoun puppy (puppy #4). No abnormalities in the aortic wall structure can be seen. Segmental hypoplasia of the muscular layer can be appreciated in the ductal wall, as evidenced by thinner media to the right of the lumen than to the left. Magnification 2×, hematoxylin and eosin stain.

In five of the eight Stabyhoun puppies, gross pathologic examination revealed a severe segmental tubular hypoplasia of the preductal segment of the aorta, i.e., cranial to the insertion of the ductus arteriosus (Figure 1 and Figure 2). In addition, the ductus arteriosus was patent in all puppies (Figure 3). No intracardiac shunts or abnormal heart valves were found in any of the dogs. The eight puppies originated from six different litters, and the five puppies with preductal segmental aortic hypoplasia originated from four different litters. No familiar relation among the litters was known, other than that all puppies originated from the Netherlands, where the Stabyhoun is considered a rare breed.

The medical histories, clinical findings, gross pathologic and focused histologic findings of the two puppies that showed clinical signs before their death are described as follows.

Puppy #1 was a clinically healthy pup when, on the 4th day of life, one of its littermates died of an unknown cause. This puppy was not submitted to necropsy. The following day, on day 5, puppy #1, was presented to the local veterinarian because of weight loss, moaning, a tense abdomen and a rectal temperature of 34 degrees of Celsius. A herpes viral infection and possible constipation were suspected, and the puppy was treated with carprofen, ampicillin and an enema. The following day puppy #1 died spontaneously and was sent to pathology the same day. The necropsy showed moderate subcutaneous edema. The lungs were moderately collapsed, pink and soft on palpation and showed a few well-defined noduli of 1–2 mm. The mediastinum and pericardium were moderately edematous, and the thorax was filled with a moderate amount of transudate. The ductus arteriosus was patent and had a larger diameter than that of the aorta. The abdominal cavity contained 6 mL of red, nontransparent fluid. The liver showed several poorly demarcated pale areas of about 5 mm in diameter. Histopathology of the lungs revealed thickened alveolar septa with a moderate number of macrophages and fibroblasts, as well as multifocal to confluent moderate atelectasis, suggestive of pulmonary edema. However, a cardiogenic origin of this suspected edema was thought to be unlikely since the left atrium and left ventricle had normal dimensions. The liver showed signs of mild to moderate extramedullary hematopoiesis, compatible with age. No changes were found that would be compatible with the clinically suspected herpes viral infection.

Puppy #4 was euthanized because of respiratory distress. The thorax contained 2.5 mL of clear red fluid and small numbers of blood clots, consistent with euthanasia by intrathoracic injection. The trachea contained a moderate amount of white foam. The lungs were moderately consolidated, pink to pale red and moist on the cutting surface, consistent with congestion. The caudal lung lobes showed slightly increased consistency. The ductus arteriosus was patent with a lumen diameter of 3–4 mm (Figure 3). The lumen of the aorta at the opening of the PDA was 2–3 mm in diameter. The moderately developed lung tissue was fairly cellular, with often slightly collapsed alveolar septa, making it difficult to determine the nature and location of the cells. There seemed to be some proliferation of type II alveolar epithelial cells, moderate proliferation of foamy alveolar macrophages and the presence of fibro-histiocytic cells in the alveolar septa.

## 4. Discussion

The present study showed a surprisingly high frequency of preductal segmental tubular aortic hypoplasia among Stabyhoun puppies that were either stillborn, died or were euthanized in the perinatal period and were submitted for necropsy to the reporting institution. The relatively high rate of occurrence of this congenital anomaly in the examined puppies suggests a genetic background in the Stabyhoun breed; however, the prevalence of disease in the population remains unknown. Identifying this congenital vascular anomaly in these puppies was a coincidental finding, and the reason why it was found is that the pathologists specifically looked for a PDA in each puppy. 

Though aortic coarctation, a form of preductal segmental tubular aortic hypoplasia, is a relatively common congenital anomaly in human infants, it has rarely been reported in animals, including dogs [4,5,6,7,8,9,10,11,12,13,14]. Because of the high reported prevalence of PDA in the Stabyhoun breed and its suspected potential role in the high perinatal mortality before the start of this study, special attention was paid to the patency of the ductus arteriosus and the surrounding vascular structures at necropsy [3]. Dissecting the intrathoracic aorta and the aortic arch in newborn puppies at post-mortem examination is challenging because of the small size; therefore, it is not routinely performed [17]. Consequently, aortic coarctation and segmental tubular aortic hypoplasia might be underdiagnosed, as they might be more common in stillborn and perinatally deceased puppies than reported so far. To gain more information about this condition in animals, dissection of the aortic arch and intrathoracic aorta should become a routine part of necropsy especially in stillborn and perinatally deceased puppies.

Coarctation of the aorta is a stenosis of the aortic isthmus, which is located at the level of the insertion of the ductus arteriosus [6,7,8,9,10]. The underlying mechanism of the development of this condition is thought to be the contraction of ectopic ductal smooth muscle in the aortic wall at birth [6,9]. This theory is supported by the observation that clinical improvement occurs after prostaglandin administration in severely affected human neonates, which treatment prevents physiologic contraction of the ductus arteriosus and the ectopic ductal tissue [9]. Stenosis of the aorta can also occur at other anatomical segments than the juxtaductal region [4,5,6,7,8,9,10].

Clinical signs of aortic coarctation appear only after birth because, in the fetus, most of the cardiac output from the right ventricle bypasses the lung and enters the descending aorta via the ductus arteriosus [9]. Because only a small amount of combined ventricular output flows across the aortic isthmus, fetal survival is not compromised in cases of a narrow aortic segment at this level [9]. Clinical signs appear only after closure of the foramen ovale and the ductus arteriosus, when the left ventricular output must flow through the narrowed aorta [9]. A sudden increase in the left ventricular afterload results in decreased left ventricular stroke volume and increased left ventricular end-diastolic and left atrial pressures [9]. As an end result, shock and cardiogenic pulmonary edema develop, leading to poor peripheral perfusion and tachypnea with respiratory distress around 7 days of life in human infants [9]. Without intervention, multiorgan failure and death are inevitable [9]. If the coarctation is less severe, systemic arterial hypertension and, as a consequence, ischemic or hypertensive organ damage occur [9]. Characteristic cardiovascular changes of affected human patients are left ventricular concentric hypertrophy and development of arterial collaterals via the subclavian and intercostal arteries that bypass the area of stenosis and provide blood flow to the descending thoracic aorta [9]. Aortic coarctation in adults can lead to death through congestive left-sided heart failure, aortic rupture, bacterial endocarditis or intracranial hemorrhage [9].

It is unlikely that the same pathogenesis was responsible for the preductal segmental tubular aortic hypoplasia in the stillborn Stabyhoun puppies as the proposed mechanism in humans because constriction of the ectopic ductal muscle in the aortic wall would only take place after live birth. Consequently, aortic hypoplasia could not be the cause of intrauterine death either. Another theory for the pathogenesis of aortic coarctation in humans proposes the presence of low flow through the aortic isthmus in the intrauterine period in cases of a large intracardiac shunt, typically a ventricular septal defect [9]. Because in none of the five reported Stabyhoun puppies was an intracardiac congenital heart disease found, this theory cannot explain the pathogenesis either. Recent human studies have suspected that a diffuse aortopathy might play a role in the pathogenesis or a genetic background without a clearly defined etiology in fetal aortic coarctation [10].

Patency of the arterial duct in stillborn puppies is not a surprising finding at necropsy, as ductal closure takes place only after birth [1,18]. Though the ductus arteriosus functionally closes on the day of birth in dogs, at necropsy, it can be patent until 1 month of age [18]. Therefore, diagnosing PDA with certainty in dogs that died or were euthanized during the first month of their lives can be difficult, especially in the absence of macroscopic signs of left ventricular volume overload or pulmonary hypertension. However, histopathology can confirm segmental muscular hypoplasia in the wall of the ductus arteriosus [19]. Because preductal narrowing of the aorta has no adverse effects on the circulation in a fetus, this condition cannot be responsible for the death of the stillborn puppies in our case series. The puppy that was euthanized because of respiratory distress might have suffered from congestive heart failure due to the additive hemodynamic consequences of the simultaneously present PDA and preductal segmental tubular aortic hypoplasia. However, the histopathologic findings of the lungs were most likely caused by pneumonia, as cardiogenic pulmonary edema can be ruled out by the lack of left atrial dilatation and left ventricular hypertrophy. Whether the PDA and the preductal aortic tubular hypoplasia in the Stabyhoun puppies are genetically related disorders remains to be determined.

A major limitation of this study is the lack of detailed anatomical descriptions with consistent measurements of the aortic arch and the ascending aorta. The reason for this shortcoming is that the present study was meant to be a pilot study, with a planned follow-up with a larger number of puppies. Unfortunately, the project stopped for an unknown reason since no Stabyhoun puppies were submitted for necropsy over the past 5 years. Another limitation of the study is that the litter sizes and the circumstances of birth, such as preterm or prolonged birth, were not available for each dog. Additionally, no clinical examination findings, such as the presence of a murmur, from the surviving littermates were available.

## 5. Conclusions

Post-mortem examination revealed an unusually high frequency of preductal segmental tubular aortic hypoplasia in Stabyhoun puppies that were either stillborn, died or were euthanized before 3 weeks of age and were submitted for necropsy. The present study aims to raise awareness of this rarely documented congenital vascular anomaly in dogs, which might have been overlooked so far because dissection of the large vessels is not routinely performed at necropsy, especially in perinatally deceased puppies.

## Figures and Tables

**Table 1:** Characteristics of the five individual Stabyhoun puppies with preductal segmental tubular hypoplasia of the aorta.

	Sex	Weight at Necropsy	Birth	Clinical Signs	Death	Known Data on the Litter
#1	female	440 g	normal	on day 5, non-specific signs	spontaneous death on day 6	largest pup of a litter of 6 pups
#2	female	220 g	dead	not applicable	stillborn	littermate of #3
#3	male	270 g	normal	palatoschisis 2 cm of the hard and soft palate	euthanasia on day 1	littermate of #2
#4	male	750 g	normal	dyspnea	euthanasia on day 16	litter of 10 pups
#5	male	210 g	dead	not applicable	stillborn	lightest pup of the litter

## Data Availability

Data are available from the corresponding author upon reasonable request.
